# The timing of cognitive plasticity in physiological aging: a tDCS study of naming

**DOI:** 10.3389/fnagi.2014.00131

**Published:** 2014-06-24

**Authors:** Anna Fertonani, Michela Brambilla, Maria Cotelli, Carlo Miniussi

**Affiliations:** ^1^Cognitive Neuroscience Section, IRCCS Istituto Centro San Giovanni di Dio FatebenefratelliBrescia, Italy; ^2^Neuroscience Section, Department of Clinical and Experimental Sciences, University of BresciaBrescia, Italy

**Keywords:** aging, language, transcranial direct current stimulation, facilitation, neuroplasticity, NIBS

## Abstract

This study aimed to explore the effects of transcranial direct current stimulation (tDCS) on physiologically aging adults performing a naming task. tDCS is a method that modulates human cortical excitability. Neuroplasticity is considered to have its foundation in cortical excitability as a property that adjusts the connection strength between neurons in the brain. Language efficiency, as all functions, relies on integration of information (i.e., effectiveness of connectivity) through neurons in the brain. So the use of tDCS, to modulate cortical excitability, can help to define the state of cognitive plasticity in the aging brain. Based on Hebb's rule, an increase in synaptic efficacy does not rely only on the increase of excitability but also on the timing of activation. Therefore, a key issue in this study is the timing of tDCS application in relation to a task: When to deliver tDCS to induce modulatory effects on task execution to facilitate naming. Anodal tDCS was applied to the left dorsolateral prefrontal cortex of older and young adults before and during a naming task. In older adults, tDCS improved naming performance and decreased the verbal reaction times only if it was applied during the task execution, whereas in young subjects both stimulation conditions improved naming performance. These findings highlight that in healthy aging adults, the cerebral network dedicated to lexical retrieval processing may be facilitated only if stimulation is applied to an “active” neural network. We hypothesize that this change is due to the neuronal synaptic changes, in the aging brain, which reduce the window of when cortical excitability can facilitate synaptic efficacy and therefore plasticity.

## Introduction

Language is a critical cognitive function for the communication processes in humans, and it is vital to successful social functioning. In general, the effect of physiological aging on language is characterized by a complex pattern of alterations (Cotelli et al., 2012), and evidences suggest that normal aging selectively impairs certain language abilities more than others. Although older adults know more words than young adults (Kemper and Sumner, 2001), they are more likely to experience difficulty that is manifested as slowing in producing words while speaking (Kemper, 2006; Burke and Shafto, 2008). Notable declines in these kinds of language processing undermine older adults' abilities and desire to communicate which promotes withdrawal from social interaction (Hummert et al., 2004), that might favor a more general cognitive decline (Palmer, 1990). Conversely, the beneficial role of an enriched environment in promoting the plasticity in aging has now been demonstrated. Studies on aged rodents showed that an enriched environment increases neurogenesis (Speisman et al., 2013), and significantly improved the rate and extent of stroke recovery (Buchhold et al., 2007). Studies focusing on word production by employing picture-naming tasks have demonstrated an age-related decline in object and action naming (Goodglass, 1980; Nicholas et al., 1985; LaBarge et al., 1986; Ardila and Rosselli, 1989; Feyereisen, 1997). Among others, a work by Goral et al. (2007) supports the hypothesis that the word retrieval declines, during healthy aging (Burke and Mackay, 1997; Thornton and Light, 2006; Burke and Shafto, 2008) suggesting an important role of prefrontal structures in these tasks (Cabeza, 2002).

Recent evidences have suggested that brain modifications underlying the decline of cognitive functions (associated with physiological aging) are not caused by neuronal loss; rather, they are associated with alterations in synaptic connectivity (Pakkenberg et al., 2003; Morrison and Baxter, 2012). These alterations are more pronounced in anterior than in posterior brain regions, and they reach a maximum level in the prefrontal cortex (Davis et al., 2008). One influential hypothesis focuses on the role of the dendritic spines at the dorsolateral prefrontal cortex (DLPFC) level; the hypothesis specifically references thin spines, whose numbers are apparently greatly reduced during healthy aging (Peters et al., 2008; Dumitriu et al., 2010). Therefore, the efficacy of cognitive performance and plasticity mechanisms may be altered during healthy aging.

Transcranial direct current stimulation (tDCS) is a technique that utilizes the application of a very low direct current (Priori, 2003; Nitsche et al., 2008; Paulus, 2011). During the delivery of the current (i.e., online stimulation), tDCS modulates the resting membrane potential of neurons in a direction that depends on the polarity (anodal vs. cathodal) of the electrode placed on the chosen area. Since the first studies (Nitsche and Paulus, 2001; Nitsche et al., 2003b), the presence of offline effects (effects that persist beyond the stimulation period) has been highlighted in addition to the online effects (during stimulation). Online and offline effects seem mediated by different mechanisms. It has been showed that online anodal tDCS-induced effects are related to membrane depolarization because they are effected by ion-channel blocking substances (Stagg and Nitsche, 2011), while offline tDCS-induced effects involve the additional participation of glutamatergic N-methyl-D-aspartic (NMDA) receptors and therefore a long-term potentiation-like (LTP-like) mechanism (Liebetanz et al., 2002; Stagg and Nitsche, 2011). Therefore, changes in duration of the induced effect should depend on change on one of these mechanisms.

Several studies have recently applied anodal tDCS with the aim of highlighting the presence of behavioral facilitator effects on several cognitive functions including language (see Monti et al., 2012 for a recent review). For example, Fertonani et al. (2010) observed a behavioral facilitation in a naming task after the application of anodal tDCS on the left DLPFC. This facilitatory function performed by tDCS may be important in cognitive neurorehabilitation approaches because the technique can improve reduced cognitive abilities in several neurological pathologies. Nevertheless, one caveat is that most of these studies were conducted on young healthy adults, and the generalization of the effects to other subjects (particularly patients who are not in the same age range of younger adult subjects) is not guaranteed (see Holland and Crinion, 2012). From this perspective, the physiological aging brain represents an interesting model to study and to predict result of tDCS applications in the clinical field; generally, patients that enter a neurorehabilitation approach are not young and therefore their brain is similar to the brain of a healthy older adult. Understanding what happens when tDCS is applied to an aged brain would validate the application of transcranial electric stimulation methods in a population that could maximally benefit from the enhancement of residual abilities.

For these reasons, researchers have recently become interested in the application of tDCS to older adults (Holland et al., 2011; Ross et al., 2011). Nevertheless, some issues must be clarified before using tDCS in rehabilitative protocols. An aspect that has not been adequately investigated in previous studies is the optimal time to apply tDCS when testing cognitive function. Some recent studies have suggested that when to deliver tDCS in relation to a task of interest may represent a key variable in determining the effects of tDCS (Stagg et al., 2011; Pirulli et al., 2013, 2014). An interesting work by Wirth et al. (2011) examined the behavioral and electrophysiological effects of online and offline anodal tDCS during a naming task. The behavioral effect was present only with online application. After anodal offline tDCS there was not behavioral effect but was present a reduction in delta activity, interpreted as a sign of neural disinhibition.

If neuroplasticity is reliant on the degree of neuronal excitability in a given moment as an element that adjusts the strength of neuronal connections in the brain, the timing of the tDCS application and when this “treatment” induces neuronal effects on task execution are important issues. The present work aims to explore the effects of tDCS in healthy aging adults performing picture-naming tasks by investigating the ideal moment for applying anodal stimulation (i.e., during or before the execution of the task). In general, tDCS may “prime” the system by (i) increasing excitability by modifying the synaptic “weights” of the system via LTP-like mechanisms that imply the involvement of NMDA receptors or (ii) increasing the excitability of the stimulated area by modulating axon, dendrite, and soma resting potentials and eventually increasing the quantity of neurotransmitters released from the presynaptic neuron without inducing long-term effects. Both of these hypotheses predict facilitation under specific conditions. The first hypothesis suggest that tDCS can induce facilitation mainly during offline applications via LTP-like mechanisms, but also during online stimulation via membrane depolarization (Liebetanz et al., 2002). The second hypothesis suggests facilitation only during online tDCS. We hypothesize that in the healthy aging brain, the mechanisms of plasticity subtending offline stimulation effects may be altered due to neuronal dysfunction (Pakkenberg et al., 2003; Morrison and Baxter, 2012). Consequently, the offline facilitation effects of tDCS should be less pronounced than the online effects. We also tested a group of young healthy subjects to investigate the possibility of different behavioral effects, induced by a different neural efficiency, in the two groups of age.

## Methods

### Subjects

Twenty young subjects (10 males; mean age 21.2 years, standard deviation 0.9, range 20–23, mean education 13.0 years) participated in the Experiment on young. Twenty healthy-aging adults (10 males, mean age 66.5 years, standard deviation 5.5, range 61–83, mean education 10.5 years) participated in the Experiment on elderly.

The subjects were right-handed native Italian speakers with normal or corrected-to-normal vision. We did not include subjects who had a history of seizures, implanted metal objects, heart problems or any other neurological or psychiatric disease. Healthy aged individuals who scored below 27 out of 30 on the Mini Mental State Examination (MMSE) were also excluded. In addition, the elderly participants were subjected to a complete, accurate neuropsychological evaluation; a pathological score in at least one of the tests was a further exclusion criterion. The neuropsychological test battery included measures to assess non-verbal reasoning (Raven Colored Progressive Matrices), language comprehension (Token test), verbal fluency (phonemic and semantic), the object-/action-naming and comprehension subtests of the Battery for the Analysis of the Aphasic Deficit (BADA) (Miceli et al., 1994), memory (Story Recall, Rey–Osterrieth Complex Figure recall, Digit Span, Spatial Span), visuo-spatial abilities (Rey–Osterrieth Complex Figure, copy), attention and executive functions (Trail-Making test A and B). The results of the cognitive assessment are presented in Table 1.

**Table 1:** **Neuropsychological data (mean) of the elderly participants**.

	**Raw score**	**Cut-off**
**SCREENING FOR DEMENTIA**		
MMSE^a^	29.0/30	24
**NON-VERBAL REASONING**		
Raven colored progressive matrices	29.5/36	17.5
**MEMORY**		
Story recall	14.1/28	7.5
Rey-Osterrieth complex figure, recall	14.2/36	9.46
Digit span	5.9	3.75
Spatial span	5.1	3.55
**PRAXIA**		
Rey–Osterrieth complex figure, copy	32.1/36	28.87
**EXECUTIVE FUNCTIONS**		
Trail-making test A (seconds)	36.4	93
Trail-making test B (seconds)	108.2	282
**LANGUAGE**		
Token test	34.0/36	26.5
Fluency, phonemic	39.1	16
Fluency, semantic	47.3	24
Oral object comprehension (BADA^b^)	39.9/40	
Oral action comprehension (BADA^b^)	19.8/20	
Oral object naming (BADA^b^)	29.5/30	
Oral action naming (BADA^b^)	27.6/28	

a Mini mental state examination

b Battery for the analysis of the aphasic deficit.

The study was approved by the Ethics Committee of IRCCS Centro San Giovanni di Dio Fatebenefratelli, Brescia, Italy. Informed consent was obtained from all participants prior to the beginning of the experiment.

### Picture-naming task

The stimuli for the picture-naming task were presented on a personal computer screen using the Presentation v. 12.0 software program (http://www.neurobs.com). All of the stimuli were black-and-white, two-dimensional line drawings from the corpus of the CRL-IPNP (http://crl.ucsd.edu/~aszekely/ipnp), a broad set of 795 action and object pictures. These items have been tested in healthy and patient populations in seven different international sites and languages. The items are coded for a number of variables known to influence naming difficulty, including initial word frequency, age of acquisition and picture imageability scores. These variables have been previously tested to assess their influence on the participants' naming performance (Bates et al., 2000).

The picture-naming task consisted of a practice block and three experimental blocks. The practice block included three object and three action images. Each experimental block included 14 object and 14 action images selected from a larger data set, which were tested in previously published behavioral experiments (Cotelli et al., 2010). We have constructed two different version of the task, to be used accordingly to the age of the experimental subjects (young, or elderly). It has been demonstrated that elderly subjects are slower than young subjects in naming pictures (Burke and Shafto, 2004). In the elderly task both the duration of stimulus presentation and the inter-stimulus interval were longer. This could potentially have affected our results slowing down the reaction times in the aging group (see e.g., Nakata et al., 2005), nevertheless, the presence of a sham condition (see below) assures us the possibility to verify if the effect is due to the stimulation or to this variable. The frequencies and lengths of the target words and the visual complexity and imageability of the pictures were matched between the blocks.

**Figure 1:**
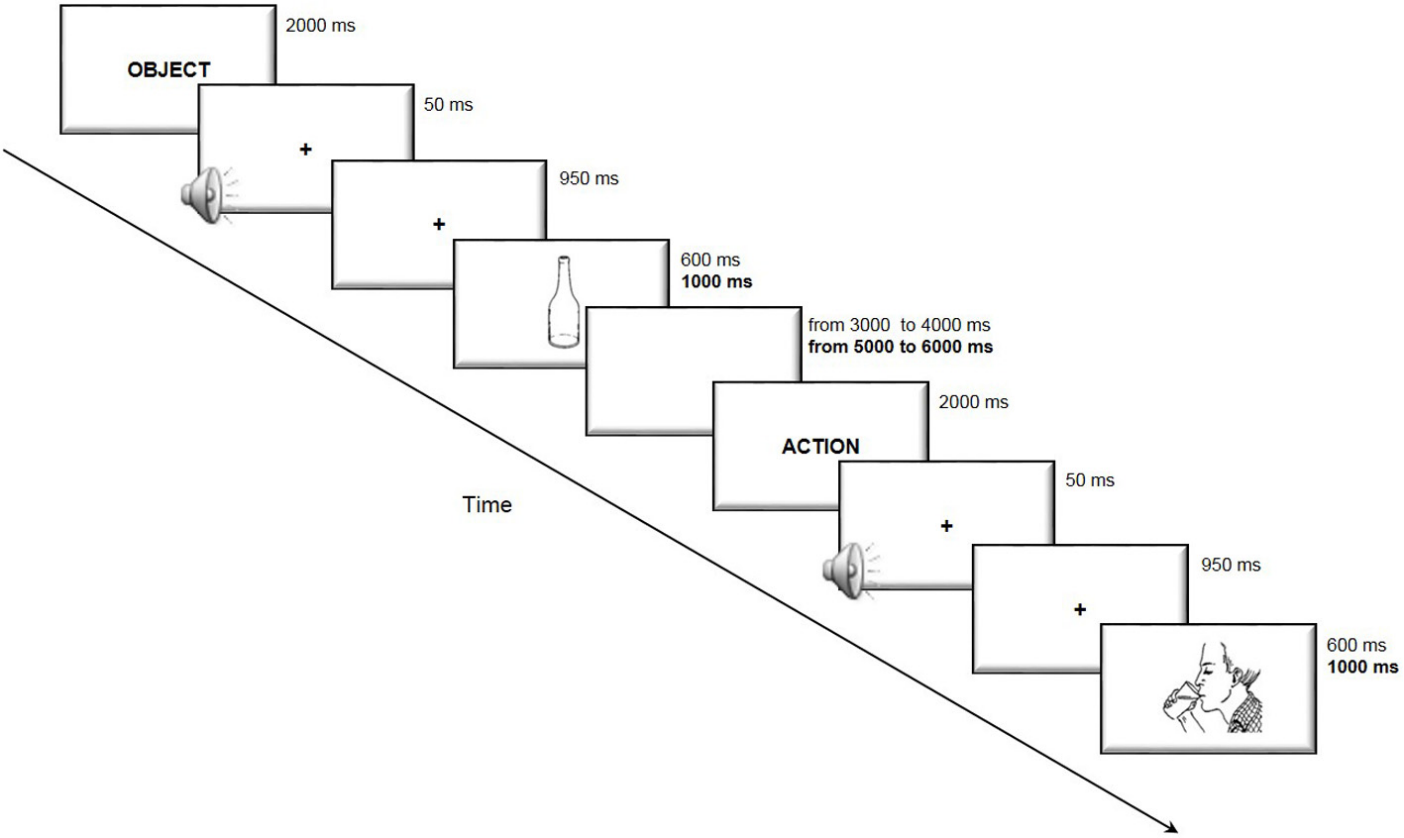
**Trial structure of the picture-naming task**. The subjects were presented with an indication of the category “action” or “object” immediately before the picture was presented to disambiguate lexical selection. The participants were then required to accurately and rapidly name the stimuli appearing on the computer screen. In bold are reported the timing used in the elderly participants task version, when different from that of the young participants.

The subjects were required to accurately and rapidly name the stimuli appearing on the computer screen. The duration of presentation of each image and of the interval between the trials was longer for elderly subjects to obtain a comparable task difficulty in the two age groups. The trial structure is illustrated in Figure 1.

### Transcranial direct current stimulation

The stimulation was delivered by a battery-driven, constant-current stimulator (BrainStim, EMS, Bologna, Italy) through a pair of saline-soaked sponge electrodes (7 × 5 cm). A constant current of 2 mA was applied with a ramping period of 10 s both at the beginning and at the end of the stimulation. In the online condition, the current was turned on at the beginning of the task and off at the end of the task, resulting in four or 5-min-long stimulation (respectively for young and elderly participants, see Figure 1). In the offline condition, the stimulation duration was set to 10 min to assure the permanence of the stimulation effects during the execution of the subsequent naming task. The current density (0.057 mA/cm^2^) was maintained below the safety limits (Poreisz et al., 2007; Nitsche et al., 2008). The electrodes were firmly attached by elastic bands, and an electroconductive gel was applied under the electrodes to reduce contact impedance before the montage. The active electrode was placed on the left DLPFC, 8 cm frontally and 6 cm laterally with respect to the scalp vertex (Fertonani et al., 2010), which had been identified as CZ in 10–20 nomenclature for EEG electrode positioning. The reference electrode was fixed on the right shoulder. We preferred an extracephalic reference to avoid interference effects from brain areas beneath the reference electrode.

The study was a single-blind experiment; the individual subjects were unaware of the type of stimulation they received, but the experimenter knew the type of stimulation. We applied the three following different types of stimulation to the left DLPFC: anodal online, anodal offline and sham. The duration of the stimulation in Experiment on young and in Experiment on elderly was respectively: 4 or 5 min for the anodal online condition (according to the different duration of the task on the two experiments), 10 min for the anodal offline condition, 6 or 7 min for the sham condition (beginning always 2 min before the beginning of the task). The different durations were due to the duration of task execution, faster in the Experiment on young (see Figure 1). In the sham stimulation (i.e., placebo), the current was turned off 10 s after the beginning of the stimulation (plus the duration of the fade-in and fade-out periods = 10 s) and was turned on for the last 10 s of the stimulation period. Therefore, the subjects felt the itching sensations below the electrodes at the beginning and end of the stimulation, making this condition indistinguishable from the experimental stimulation. Indeed, to detect any differences in the perception of sensations, we asked the subjects to complete a questionnaire developed by our research group (Fertonani et al., 2010) regarding the sensations that they experienced during the different stimulation types (real vs. sham) (See Results).

### Procedure

**Figure 2:**
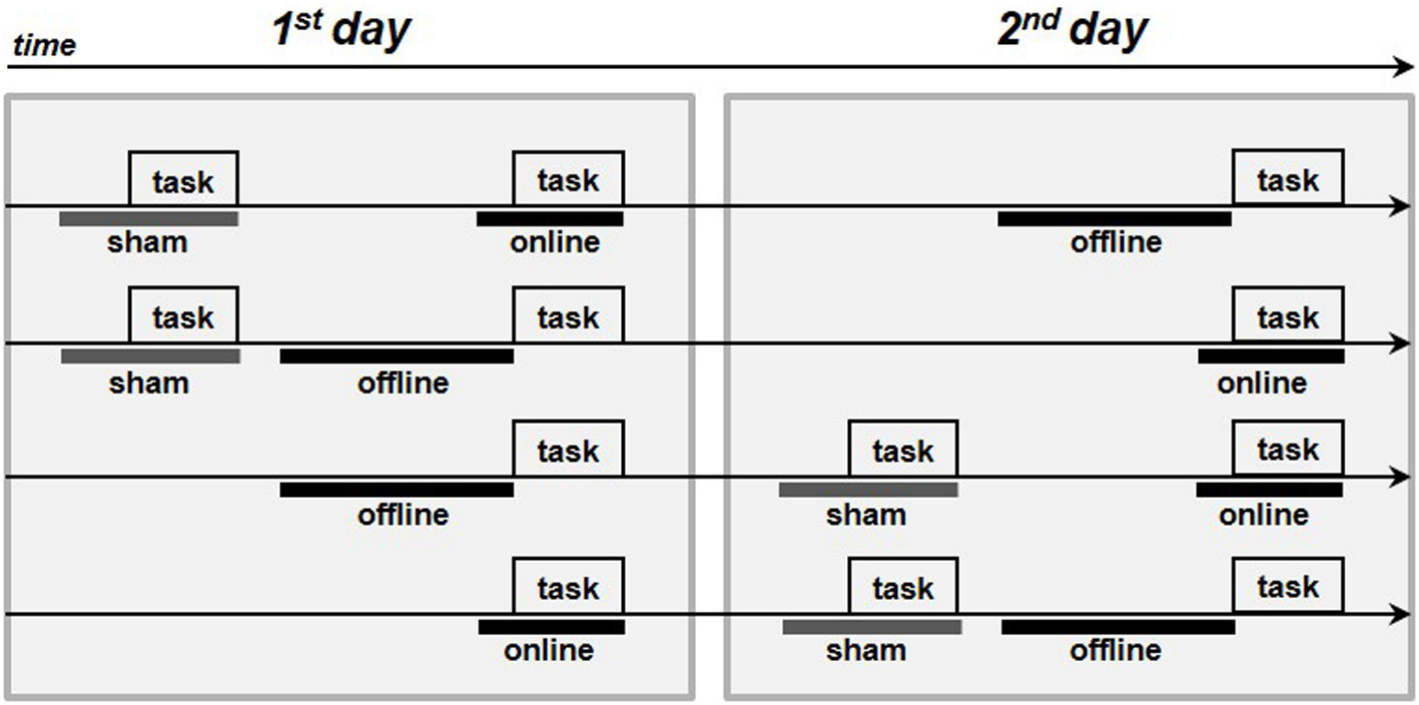
**Procedure of the experiments**. The four arrows represents the four procedures adopted in each experiment. The gray boxes represent the execution of the picture-naming task, the thick gray lines represent the sham stimulation whereas the thick black lines the real tDCS stimulations (online or offline).

The subjects were seated in a quiet room in front of a computer screen. In the anodal online condition, the subjects performed the picture-naming task during the stimulation. In the anodal offline condition, the subjects performed the task immediately following the stimulation, and in the sham condition, the placebo stimulation began approximately 2 min before the start of the task. The active stimulations (i.e., anodal online and anodal offline) were executed on two different and consecutive days to minimize the likelihood of interference effects. The sham stimulation was always performed first on the first or second day (see Figure 2).

### Data analysis

The latency of verbal responses (vocal reaction time, vRT) and naming accuracy were determined for each subject in each condition. The subjects' answers were recorded with a microphone placed in front of the participant. The vocal responses were digitized with the GoldWave v. 5.15 software program (GoldWave, Newfoundland, Canada) with a sampling rate of 11,025 Hz. The latency of the vRT was measured by analysing the GoldWave registration files, which marked the start of the wave corresponding to the vocal response. As standard procedure we eliminated all incorrectly performed trials from the analysis, including no responses, semantic errors, visual errors and responses preceded by verbal searching, (overall 3.2%, that is within the normal range). In addition, we removed all vRT data above or below two standard deviations with respect to the mean for each subject in each condition.

For the vRT data, the Kolmogorov–Smirnov test confirmed the normality of the distribution; therefore, the data were subsequently analyzed using a repeated measure ANOVA. The Mauchly test was applied when appropriate to confirm the data sphericity. Multiple comparisons were carried on using the Fisher LSD procedure.

Because the naming accuracy data were not normally distributed, we applied the appropriate non-parametric tests. A *p*-value of 0.05 was considered significant for all statistical analyses except for the sensations (see below).

The data of the tDCS-induced sensations were analyzed using the Friedman test (non-parametric repeated measures comparison). The *post-hoc* comparisons were conducted considering a α-value of 0.0167 (0.05 divided by 3, i.e., the number of comparisons performed for each sensation).

## Results

We inferred that all of the subjects tolerated the stimulation by interpreting the spontaneous reports and the questionnaires completed by each subject at the end of the experiment. As evinced from verbal reports, none of the subjects could distinguish the sham from the real stimulation. The questionnaire results are reported in Table 2. In *young subjects*, pitching, itchiness and burning were the most commonly reported sensations (90, 83, and 57% of the subjects, respectively), with light to moderate intensity. The Friedman test highlighted a statistically significant difference between the stimulations for the perception of itchiness [χ^2^_(2)_ = 11.472, *p* = 0.003], pinching [χ^2^_(2)_ = 10.750, *p* = 0.004], iron taste [χ^2^_(2)_ = 6.645, *p* = 0.04] and effect on performance [χ^2^_(2)_ = 20.167, *p* < 0.001]. Nevertheless, *post-hoc* comparison showed a significant difference only between anodal online and sham condition in the itchiness, pinching and effect on performance perception (i.e., sensations greater in the anodal online condition), and between online and offline condition in the effect on performance perception.

**Table 2:** **Mean intensity of the sensations reported by the subjects after transcranial direct current stimulation (tDCS) and the percentage of subjects who reported a certain sensation**.

		**Itchiness**	**Pain**	**Burning**	**Heat**	**Pinching**	**Iron taste**	**Fatigue**	**Effect on performance**
	**SHAM**								
	Intensity	1.0	0.2	0.9	0.4	1.4	0.2	0.1	0.1
	Subjects (%)	80	15	55	35	90	10	10	10
**Young subjects**	**ANODAL OFFLINE**								
	Intensity	1.2	0.2	0.7	0.3	1.4	0.6	0.2	0.1
	Subjects (%)	75	20	55	25	80	50	15	10
	**ANODAL ONLINE**								
	Intensity	1.8	0.4	1.0	0.4	2.0	0.4	0.2	0.7
	Subjects (%)	95	30	60	30	100	20	15	65
	**SHAM**								
	Intensity	0.3	0.0	0.4	0.1	0.8	0.1	0.0	0.0
	Subjects (%)	26	0	37	5	74	11	0	0
**Elderly subjects**	**ANODAL OFFLINE**								
	Intensity	0.3	0.0	0.4	0.1	0.9	0.1	0.0	0.1
	Subjects (%)	26	0	21	5	74	11	0	5
	**ANODAL ONLINE**								
	Intensity	0.3	0.0	0.5	0.0	0.8	0.1	0.0	0.1
	Subjects (%)	21	0	37	0	68	11	0	5

The sensation intensity is presented on a 5-point scale as follows: 0 = None, 1 = Mild, 2 = Moderate, 3 = Considerable, 4 = Strong. The column “Effect on performance” indicates the subjective feeling of the participant relative to how much the tDCS-induced sensations affected his performance.

In *elderly subjects*, light-intensity pitching and burning were the most commonly reported sensations (72 and 32% of the subjects, respectively). The Friedman test did not reveal any significant difference in the subjects' perceptions of sensation between the experimental (online or offline) or sham stimulation conditions.

### Accuracy

The accuracy level of *young participants* was high (96% mean accuracy in all of the conditions, see Table 3 for more details). The accuracy did not differ among the stimulation conditions (sham, anodal online, anodal offline)[Friedman test (χ^2^_(2)_ = 1.333, *p* = 0.51)]. The Wilcoxon test on the type of stimulus (object, action) was significant (*Z* = 3.114, *p* = 0.002). Young subjects were more accurate in the object-naming task (mean = 0.15 errors/block) than in action-naming task (mean = 0.83 errors/block).

**Table 3:** **Verbal reaction time (vRT) and accuracy for young and elderly subjects in the three experimental conditions**.

		**Sham**	**Anodal online**	**Anodal offline**
	**vRT (MS)**			
	Actions	757 ± 72	720 ± 69	710 ± 72
	Objects	585 ± 57	578 ± 55	576 ± 56
**Young subjects**	**ACCURACY (%)**			
	actions	93.6 ± 6.9	93.6 ± 6.5	95.0 ± 6.6
	objects	98.6 ± 3.7	99.3 ± 2.2	98.9 ± 3.5
	**vRT (MS)**			
	actions	912 ± 95	871 ± 99	921 ± 144
	objects	718 ± 83	691 ± 65	705 ± 84
**Elderly subjects**	**ACCURACY (%)**			
	actions	94.6 ± 6.9	95.7 ± 5.6	94.6 ± 8.5
	objects	98.6 ± 2.9	98.9 ± 1.6	99.6 ± 2.6

vRT are expressed as ms ± SD, accuracy as percentage of accuracy ± SD.

Also in the *elderly group*, the accuracy level was high (97% mean accuracy in all of the conditions, see Table 3). The accuracy did not differ among the stimulation conditions (sham, anodal online, anodal offline)[Friedman test (χ^2^_(2)_ = 0.634, *p* = 0.73)]. The Wilcoxon test on the type of stimulus (object, action) demonstrated that elderly subjects were more accurate in the object-naming task (mean = 0.15 errors/block) than in action-naming task (mean = 0.72 errors/block) (*Z* = 2.840, *p* < 0.01).

The accuracy level was not different in *young* and *elderly group* [Mann-Withney test (*Z* = −1.163, *p* = 0.245)]. Given the very low number of errors, we did not perform further analyses on these data.

### Response times

We performed a repeated measures ANOVA on vRT with stimulation condition (sham, anodal online, anodal offline) and type of stimulus (object, action) as within subjects factors and age (young, elderly) as a between subjects factor. The ANOVA highlighted the main effect of age [*F*_(1, 38)_ = 47.001; *p* < 0.001; η^2^_*P*_ = 0.553], type of stimulus [*F*_(1, 38)_ = 413.339; *p* < 0.001; η^2^_*P*_ = 0.916] and stimulation condition [*F*_(2, 76)_ = 6.634; *p* = 0.001; η^2^_*P*_ = 0.149]. The interactions between stimulation condition and age [*F*_(2, 76)_ = 3.305; *p* = 0.042; η^2^_*P*_ = 0.080], and type of stimulus and age [*F*_(1, 38)_ = 7.145; *p* = 0.008; η^2^_*P*_ = 0.167] were also statistically significant.

The main effect of age demonstrates that young subjects were faster than elderly subjects (young: 654 ± 99 ms; elderly: 803 ± 139 ms). The main effect of the type of stimulus shows that subjects were faster at object naming than at action naming (objects: 578 ± 56 ms; actions: 713 ± 74 ms).

**Figure 3:**
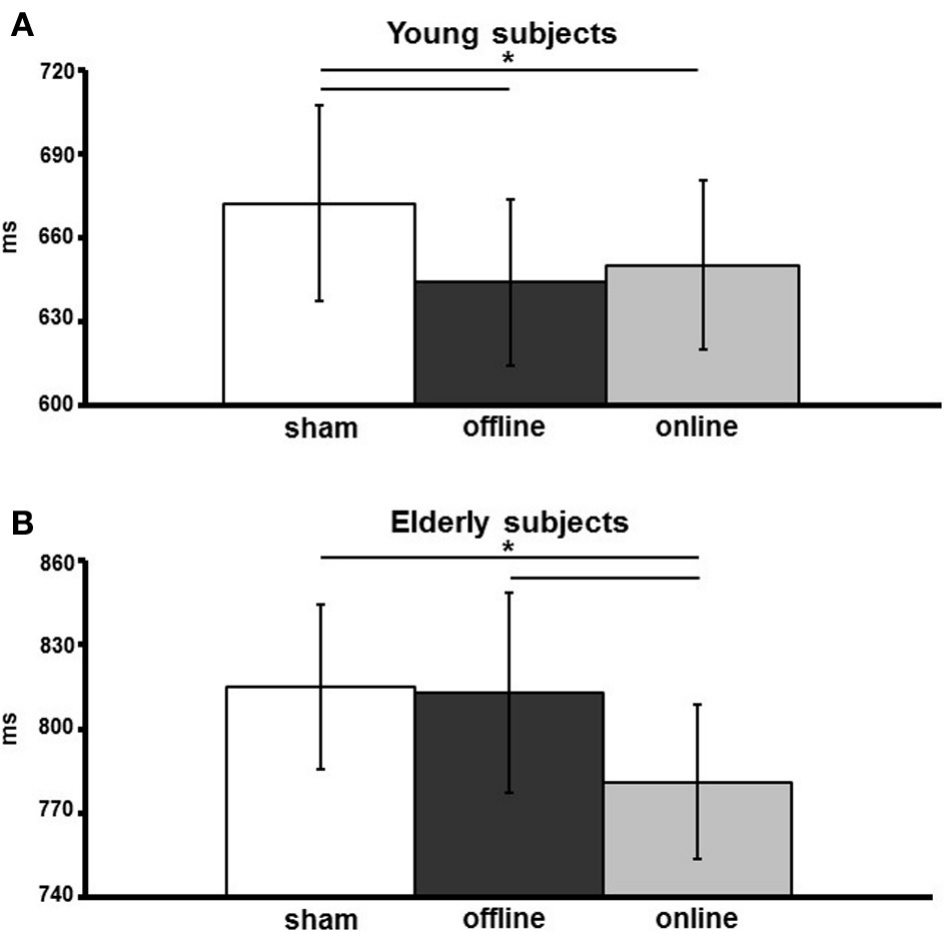
**Verbal reaction times (vRTs) of young (A) and elderly (B) participants, for the three stimulation conditions (sham, online, offline)**. The data are expressed in milliseconds along the ordinate. An asterisk indicates a *p* < 0.05. The error bars represent the mean standard error.

The effect of stimulation condition was better explained by the interaction stimulation condition × age. Multiple *post-hoc* comparisons revealed that in young there was a statistically significant difference between the sham (mean vRT ± *SD* = 671 ± 109 ms) and the anodal online (649 ± 94 ms) and anodal offline (643 ± 93 ms) conditions (respectively *p* = 0.05 and p = 0.01; see Figure 3A), whereas in the elderly there was a statistically significant difference between the anodal online condition (781 ± 123 ms) and the other two stimulation conditions (sham: 815 ± 132 ms; anodal offline: 813 ± 159 ms, both *p* < 0.01; see Figure 3B). See Table 3 for the vRT-values in each condition.

## Discussion

In this study, we show that anodal stimulation of the left DLPFC has a facilitation effect on picture naming in both young and healthy aging adults. In particular, whereas in young participants decreased vocal reaction times were present if the stimulation was applied both before and during the execution of the task, in the healthy aging group the effect was observed only if the stimulation was applied during the task execution. This result highlights the differential effects of tDCS in young and aging subjects. Moreover, it highlights the importance of stimulation timing in the aging group. Few studies to date have considered this variable only within the motor cortex (Nitsche et al., 2003a; Kuo et al., 2008; Stagg and Nitsche, 2011). Nitsche et al. (2003a) demonstrated that anodal tDCS applied during the execution of an implicit learning task led to an improvement in the learning rate of that task. However, if the same task was performed after 10 min of stimulation, no enhancement in the learning rate was observed (Kuo et al., 2008). Similar results have been reported by Stagg et al. (2011) in an explicit sequence-learning task. Online stimulation allows the subjects to learn more rapidly, and offline stimulation has the opposite effect, slowing learning. These results in the motor cortex are consistent with the hypothesis that anodal tDCS interacts with subsequent motor learning, in a metaplastic manner (Ziemann and Siebner, 2008), inducing decreased motor performance when tDCS is applied before the task. This mechanism works by reducing the amount of plasticity after an excitability-increasing stimulation (Bienenstock et al., 1982). Nevertheless, results in the motor cortex not always are comparable to tDCS effects induced in the cognitive domain (Jacobson et al., 2012).

Regarding the cognitive domain, previous studies have confirmed the presence of facilitatory effects when a cognitive task is performed after anodal stimulation (offline, e.g., Ohn et al., 2008; Sparing et al., 2008; Boggio et al., 2009; Fertonani et al., 2010), and data have highlighted analogous facilitations when the task is performed during the stimulation (Fregni et al., 2005; Chi et al., 2010). Nevertheless, because these studies did not investigate both conditions, we cannot determine whether the timing of the stimulations simply differed in the degree of induced facilitation. Moreover, all of these studies were performed on young healthy adults.

Our study compared two different application timings of anodal stimulation (i.e., online vs. offline) in the same subjects who performed the same tasks, which permits us to obtain a result that allow to directly establish if the two protocols are equivalent or not. In healthy older adults, online stimulation is better than offline in inducing facilitation when the subjects are performing a picture-naming task. However, this online vs. offline difference in inducing facilitation is inconsistent with the results in the young participants. In the present experiment and in a previous published experiment from our group (Fertonani et al., 2010) we observed a facilitation in an offline condition, confirming the validity of the effect in young subjects. Such facilitation during online and offline stimulation, in young participants is in line with previous works and therefore we need to focus on differences in the elderly.

The absence of offline facilitation in the present group of older adults may be attributed to the short stimulation period (i.e., 10 min). However, the short period should not be an issue because a stimulation of the same duration causes facilitation in young adults performing the same task, as we have demonstrated before (Fertonani et al., 2010) and confirmed in the present work. It is noteworthy that the neural networks of young and old adults are different, and the different responsiveness of healthy-aging neural networks may account for the absence of offline facilitation. The online facilitation is likely due to the action mechanisms of anodal stimulation, which acts on the neuronal membrane potential, making the stimulated neuronal population more excitable. These mechanisms are not efficacious enough, in healthy older adults, to induce short-term plasticity after stimulation. Studies have demonstrated that the aging brain is characterized by several structural, physiological and functional changes (Caserta et al., 2009). Studies on both humans and animals have proposed that age-related cognitive decline is more likely to be associated with alterations in synaptic connectivity, intracellular signaling and metabolism than with neuronal loss (Morrison and Baxter, 2012). In rhesus monkeys, researchers have demonstrated that the age-related volume decrease observed in the DLPFC is attributed to a substantial synapse loss, which involves only certain synapse types (Peters et al., 2008; Dumitriu et al., 2010). Indeed, only the thin spines are involved; these spines are associated with a high degree of plasticity compared with mushroom spines. As previously reported the offline effects of a single application of tDCS have been shown to be mediated by NMDA receptor activity, the results of an LTP-like neuronal plasticity mechanisms (Liebetanz et al., 2002; Nitsche et al., 2003a). Our data suggest that, at least with the parameters adopted in this study, the stimulation of the left DLPFC in healthy aging adults cannot induce this type of plasticity; the stimulation likely intrinsically modifies the electrical properties of the neurons. This reduced plasticity induction is presumably due to the neuronal synaptic changes that involve the aging brain. Ca^2+^ is known to be closely involved in synaptic plasticity, particularly in triggering LTP (Fitzjohn and Collingridge, 2002). Researchers have proposed that aging is associated with an increased dysregulation of Ca^2+^ homeostasis (Kirischuk and Verkhratsky, 1996). We speculate that the facilitation effect of offline tDCS may be reduced by a suboptimal intracellular Ca^2+^ level. This level may not favor the modulation of ion channel conductance and consequent homeostasis mechanisms. tDCS is a continuous stimulation; continuous stimulation can induce neurophysiological homeostasis mechanisms, which serve to maintain neural activity within a normal functional range (Siebner et al., 2004; Siebner, 2010). Present result cannot be accounted by a slowing of the system in older adults, since the facilitatory effect was present during task execution. In this context, it is possible that in older adults, the offline stimulation protocol cannot adjust the threshold of the system due to reduced transmembrane Ca^2+^ conduction. Therefore, altering the intracellular Ca^2+^ concentrations, which is important for processes of LTP, reduces tDCS-induced after-effects. Indeed Ca^2+^ alteration can also modify compensatory mechanisms of metaplasticity. Given that the synaptic homeostasis is changed so will be the degree of the response to activity induce by the task and by tDCS.

In conclusion, we found that anodal stimulation applied to the left DLPFC during the execution of a picture-naming task modulates the behavioral performance of healthy aging adults. This result confirms that the left DLPFC is part of a cerebral network dedicated to lexical retrieval/selection processing in naming (Cappa et al., 2002; Cotelli et al., 2006, 2008, 2010; Fertonani et al., 2010). Crucially, the offline tDCS application, which is efficacious in young adults, did not induce facilitation effects in older adults.

It is fundamentally important to evaluate tDCS effectiveness in inducing facilitator effects in a linguistic elaboration process to facilitate the implementation of a better informed tDCS application in the neurorehabilitation field. The current study confirmed the importance of the timing choice when applying tDCS with respect to the age of experimental subjects. tDCS-induced effects are sensitive to the state of the network that is active at that moment. It has been showed that increasing cortical excitability by means of non-invasive brain stimulation may induce a reconfiguration of functional brain networks to address specific cognitive demands (Peña-Gómez et al., 2012). Thus, the polarization of neurons in combination with ongoing synaptic input can be contextualized in a framework of synaptic co-activation (Miniussi et al., 2013). Therefore the effects of tDCS should be considered in relation to the state of the cortical network carrying out a task, network that in the aging brain might present a reduced connectivity (Sala-Llonch et al., 2014). Only in this context a-tDCS-induced modulation, might primes cortical synaptic efficacy and connectivity that potentiates the system within the language network, leading to more effective processing (Vidal-Piñeiro et al., 2014).

Based on present observations and theories (Hebb, 1949), we conclude that the capacity of an aged neural circuits to increase efficiency will be maximized when the task is executed during tDCS. Moreover, based on the same logic, learning, related to task execution, during tDCS should be more stable than learning that occurs at a different time.

### Conflict of interest statement

The authors declare that the research was conducted in the absence of any commercial or financial relationships that could be construed as a potential conflict of interest.

## References

[B1] Ardila A. Rosselli M. (1989). Neuropsychological characteristics of normal aging. Dev. Neuropsychol. 5, 307–320 10.1080/87565648909540441

[B2] Bates E. Andonova E. D'Amico S. Jacobsen T. Kohnert K. Lu C. C. (2000). Introducing the CRL International Picture-Naming Project (CRL-IPNP). Vol. 12, La Jolla, CA: Center for Research in Language Newsletter

[B3] Bienenstock E. L. Cooper L. N. Munro P. W. (1982). Theory for the development of neuron selectivity: orientation specificity and binocular interaction in visual cortex. J. Neurosci. 2, 32–48 7054394 10.1523/JNEUROSCI.02-01-00032.1982 PMC6564292

[B4] Boggio P. S. Khoury L. P. Martins D. C. S. Martins O. E. M. S. de Macedo E. C. Fregni F. (2009). Temporal cortex direct current stimulation enhances performance on a visual recognition memory task in Alzheimer disease. J. Neurol. Neurosurg. Psychiatry 80, 444–447 10.1136/jnnp.2007.141853 18977813

[B5] Buchhold B. Mogoanta L. Suofu Y. Hamm A. Walker L. Kessler C. (2007). Environmental enrichment improves functional and neuropathological indices following stroke in young and aged rats. Restor. Neurol. Neurosci. 25, 467–484 18334765

[B6] Burke D. M. Mackay D. G. (1997). Memory, language, and ageing. Philos. Trans. R. Soc. Lond. B. Biol. Sci. 352, 1845–1856 10.1098/rstb.1997.0170 9460069 PMC1692140

[B7] Burke D. M. Shafto M. A. (2004). Aging and language production. Curr. Dir. Psychol. Sci. 13, 21–24 10.1111/j.0963-7214.2004.01301006.x 18414600 PMC2293308

[B8] Burke D. M. Shafto M. A. (2008). Language and aging, in The Handbook of Aging and Cognition, eds Craik F. Salthouse T. (New York, NY: Psychology Press), 373–443

[B9] Cabeza R. (2002). Hemispheric asymmetry reduction in older adults: the HAROLD model. Psychol. Aging 17, 85–100 10.1037/0882-7974.17.1.85 11931290

[B10] Cappa S. F. Sandrini M. Rossini P. M. Sosta K. Miniussi C. (2002). The role of the left frontal lobe in action naming - rTMS evidence. Neurology 59, 720–723 10.1212/WNL.59.5.720 12221163

[B11] Caserta M. T. Bannon Y. Fernandez F. Giunta B. Schoenberg M. R. Tan J. (2009). Normal brain aging clinical, immunological, neuropsychological, and neuroimaging features. Int. Rev. Neurobiol. 84, 1–19 10.1016/S0074-7742(09)00401-2 19501710

[B12] Chi R. P. Fregni F. Snyder A. W. (2010). Visual memory improved by non-invasive brain stimulation. Brain Res. 1353, 168–175 10.1016/j.brainres.2010.07.062 20682299

[B13] Cotelli M. Manenti R. Brambilla M. Zanetti O. Miniussi C. (2012). Naming ability changes in physiological and pathological aging. Front. Neurosci. 6:120 10.3389/fnins.2012.00120 22933989 PMC3422757

[B14] Cotelli M. Manenti R. Cappa S. F. Geroldi C. Zanetti O. Rossini P. M. (2006). Effect of transcranial magnetic stimulation on action naming in patients with Alzheimer disease. Arch. Neurol. 63, 1602–1604 10.1001/archneur.63.11.1602 17101829

[B15] Cotelli M. Manenti R. Cappa S. F. Zanetti O. Miniussi C. (2008). Transcranial magnetic stimulation improves naming in Alzheimer disease patients at different stages of cognitive decline. Eur. J. Neurol. 15, 1286–1292 10.1111/j.1468-1331.2008.02202.x 19049544

[B16] Cotelli M. Manenti R. Rosini S. Calabria M. Brambilla M. Bisiacchi P. S. (2010). Action and object naming in physiological aging: An rTMS study. Front. Aging Neurosci. 2:151 10.3389/fnagi.2010.00151 21151376 PMC2996246

[B17] Davis S. W. Dennis N. A. Daselaar S. M. Fleck M. S. Cabeza R. (2008). Que PASA? The posterior-anterior shift in aging. Cereb. Cortex 18, 1201–1209 10.1093/cercor/bhm155 17925295 PMC2760260

[B18] Dumitriu D. Hao J. Hara Y. Kaufmann J. Janssen W. G. M. Lou W. (2010). Selective changes in thin spine density and morphology in monkey prefrontal cortex correlate with aging-related cognitive impairment. J. Neurosci. 30, 7507–7515 10.1523/JNEUROSCI.6410-09.2010 20519525 PMC2892969

[B19] Fertonani A. Rosini S. Cotelli M. Rossini P. M. Miniussi C. (2010). Naming facilitation induced by transcranial direct current stimulation. Behav. Brain Res. 208, 311–318 10.1016/j.bbr.2009.10.030 19883697

[B20] Feyereisen P. (1997). A meta-analytic procedure shows an age-related decline in picture naming: comments on Goulet, Ska, and Kahn (1994). J. Speech Lang. Hear. Res. 40, 1328–1333 9430752 10.1044/jslhr.4006.1328

[B21] Fitzjohn S. M. Collingridge G. L. (2002). Calcium stores and synaptic plasticity. Cell Calcium 32, 405–411 10.1016/S0143416002001999 12543099

[B22] Fregni F. Boggio P. S. Nitsche M. Bermpohl F. Antal A. Feredoes E. (2005). Anodal transcranial direct current stimulation of prefrontal cortex enhances working memory. Exp. Brain Res. 166, 23–30 10.1007/s00221-005-2334-6 15999258

[B23] Goodglass H. (1980). Naming disorders in aphasia and aging, in Language and Comunication in the Ederly, eds Obler L. K. Albert M. L. (Toronto, ON: Lexington Books), 37–45

[B24] Goral M. Spiro A. I. Albert M. Obler L. Connor L. (2007). Change in lexical-retrieval skills in adulthood. Ment. Lex. 2, 215–240 10.1075/ml.2.2.05gor

[B25] Hebb D. O. (1949). The Organization of Behavior: a Neuropsychological Theory. New York, NY: Wiley

[B26] Holland R. Crinion J. (2012). Can tDCS enhance treatment of aphasia after stroke?. Aphasiology 26, 1169–1191 10.1080/02687038.2011.616925 23060684 PMC3464450

[B27] Holland R. Leff A. P. Josephs O. Galea J. M. Desikan M. Price C. J. (2011). Speech facilitation by left inferior frontal cortex stimulation. Curr. Biol. 21, 1403–1407 10.1016/j.cub.2011.07.021 21820308 PMC3315006

[B28] Hummert M. Garstka T. Ryan E. Bonnesen J. (2004). The role of age stereotypes in interpersonal communication, in Handbook of Communication and Aging Research, eds Nussbaum J. Coupland J. (Mahwah, NJ: Routlege), 91–115

[B29] Jacobson L. Koslowsky M. Lavidor M. (2012). tDCS polarity effects in motor and cognitive domains: a meta-analytical review. Exp. brain Res. 216, 1–10 10.1007/s00221-011-2891-9 21989847

[B30] Kemper S. (2006). Language in adulthood, in Lifespan Cognition: Mechanisms of Change, eds Bialystok E. Craik F. I. M. (New York, NY: Oxford University Press), 223–238 10.1093/acprof:oso/9780195169539.003.0015

[B31] Kemper S. Sumner A. (2001). The structure of verbal abilities in young and older adults. Psychol. Aging 16, 312–322 10.1037/0882-7974.16.2.312 11405318

[B32] Kirischuk S. Verkhratsky A. (1996). Calcium homeostasis in aged neurones. Life Sci. 59, 451–459 10.1016/0024-3205(96)00324-4 8761333

[B33] Kuo M.-F. F. Unger M. Liebetanz D. Lang N. Tergau F. Paulus W. (2008). Limited impact of homeostatic plasticity on motor learning in humans. Neuropsychologia 46, 2122–2128 10.1016/j.neuropsychologia.2008.02.023 18394661

[B34] LaBarge E. Edwards D. Knesevich J. W. (1986). Performance of normal elderly on the Boston Naming Test. Brain Lang. 27, 380–384 10.1016/0093-934X(86)90026-X 3955345

[B35] Liebetanz D. Nitsche M. A. Tergau F. Paulus W. (2002). Pharmacological approach to the mechanisms of transcranial DC-stimulation-induced after-effects of human motor cortex excitability. Brain 125, 2238–2247 10.1093/brain/awf238 12244081

[B36] Miceli G. Laudanna A. Burani C. Papasso R. (1994). Batteria per l'Analisi dei Deficit Afasici. B.A.D.A. (Battery for Analysis of Aphasic Deficits). Milano: CEPSAG, Università Cattolica del Sacro Cuore

[B37] Miniussi C. Harris J. A. Ruzzoli M. (2013). Modelling non-invasive brain stimulation in cognitive neuroscience. Neurosci. Biobehav. Rev. 37, 1702–1712 10.1016/j.neubiorev.2013.06.014 23827785

[B38] Monti A. Ferrucci R. Fumagalli M. Mameli F. Cogiamanian F. Ardolino G. (2012). Transcranial direct current stimulation (tDCS) and language. J. Neurol. Neurosurg. Psychiatry 84, 832–842 10.1136/jnnp-2012-302825 23138766 PMC3717599

[B39] Morrison J. H. Baxter M. G. (2012). The ageing cortical synapse: hallmarks and implications for cognitive decline. Nat. Rev. Neurosci. 13, 240–250 10.1038/nrn3200 22395804 PMC3592200

[B40] Nakata H. Inui K. Wasaka T. Tamura Y. Kida T. Kakigi R. (2005). Effects of ISI and stimulus probability on event-related go/nogo potentials after somatosensory stimulation. Exp. Brain Res. 162, 293–299 10.1007/s00221-004-2195-4 15599719

[B41] Nicholas M. Obler L. Albert M. L. Goodglass H. (1985). Lexical retrieval in healthy aging. Cortex 21, 595–606 10.1016/S0010-9452(58)80007-6 4092486

[B42] Nitsche M. A. Cohen L. G. Wassermann E. M. Priori A. Lang N. Antal A. (2008). Transcranial direct current stimulation: state of the art 2008. Brain Stimul. 1, 206–223 10.1016/j.brs.2008.06.004 20633386

[B43] Nitsche M. A. Fricke K. Henschke U. Schlitterlau A. Liebetanz D. Lang N. (2003a). Pharmacological modulation of cortical excitability shifts induced by transcranial direct current stimulation in humans. J. Physiol. 553, 293–301 10.1113/jphysiol.2003.049916 12949224 PMC2343495

[B44] Nitsche M. A. Nitsche M. S. Klein C. C. Tergau F. Rothwell J. C. Paulus W. (2003b). Level of action of cathodal DC polarisation induced inhibition of the human motor cortex. Clin. Neurophysiol. 114, 600–604 10.1016/S1388-2457(02)00412-1 12686268

[B45] Nitsche M. A. Paulus W. (2001). Sustained excitability elevations induced by transcranial DC motor cortex stimulation in humans. Neurology 57, 1899–1901 10.1212/WNL.57.10.1899 11723286

[B46] Ohn S. H. Park C.-I. Yoo W.-K. Ko M.-H. Choi K. P. Kim G.-M. (2008). Time-dependent effect of transcranial direct current stimulation on the enhancement of working memory. Neuroreport 19, 43–47 10.1097/WNR.0b013e3282f2adfd 18281890

[B47] Pakkenberg B. Pelvig D. Marner L. Bundgaard M. J. Gundersen H. J. G. Nyengaard J. R. (2003). Aging and the human neocortex. Exp. Gerontol. 38, 95–99 10.1016/S0531-5565(02)00151-1 12543266

[B48] Palmer R. M. (1990). “Failure to thrive” in the elderly: diagnosis and management. Geriatrics 45, 47–50</page>, <page>53–5 2204587

[B49] Paulus W. (2011). Transcranial electrical stimulation (tES - tDCS; tRNS, tACS) methods. Neuropsychol. Rehabil. 21, 602–617 10.1080/09602011.2011.557292 21819181

[B50] Peña-Gómez C. Sala-Lonch R. Junqué C. Clemente I C. Vidal D. Bargalló N. (2012). Modulation of large-scale brain networks by transcranial direct current stimulation evidenced by resting-state functional MRI. Brain Stimul. 5, 252–263 10.1016/j.brs.2011.08.006 21962981 PMC3589751

[B51] Peters A. Sethares C. Luebke J. I. (2008). Synapses are lost during aging in the primate prefrontal cortex. Neuroscience 152, 970–981 10.1016/j.neuroscience.2007.07.014 18329176 PMC2441531

[B52] Pirulli C. Fertonani A. Miniussi C. (2013). The role of timing in the induction of neuromodulation in perceptual learning by transcranial electric stimulation. Brain Stimul. 6, 683–689 10.1016/j.brs.2012.12.005 23369505

[B53] Pirulli C. Fertonani A. Miniussi C. (2014). Is neural hyperpolarization by cathodal stimulation always detrimental at the behavioral level? Front. Behav. Neurosci. 8:226 10.3389/fnbeh.2014.00226 PMC4073198 25018709

[B54] Poreisz C. Boros K. Antal A. Paulus W. (2007). Safety aspects of transcranial direct current stimulation concerning healthy subjects and patients. Brain Res. Bull. 72, 208–214 10.1016/j.brainresbull.2007.01.004 17452283

[B55] Priori A. (2003). Brain polarization in humans: a reappraisal of an old tool for prolonged non-invasive modulation of brain excitability. Clin. Neurophysiol. 114, 589–595 10.1016/S1388-2457(02)00437-6 12686266

[B56] Ross L. A. McCoy D. Coslett H. B. Olson I. R. Wolk D. A. (2011). Improved proper name recall in aging after electrical stimulation of the anterior temporal lobes. Front. Aging Neurosci. 3:16 10.3389/fnagi.2011.00016 22016735 PMC3191456

[B57] Sala-Llonch R. Junqué C. Arenaza-Urquijo E. M. Vidal-Piñeiro D. Valls-Pedret C. Palacios E. M. (2014). Changes in whole-brain functional networks and memory performance in aging. Neurobiol Aging. [Epub ahead of print]. 10.1016/j.neurobiolaging.2014.04.007 24814675

[B58] Siebner H. R. (2010). A primer on priming the human motor cortex. Clin. Neurophysiol. 121, 461–463 10.1016/j.clinph.2009.12.009 20064742

[B59] Siebner H. R. Lang N. Rizzo V. Nitsche M. A. Paulus W. Lemon R. N. (2004). Preconditioning of low-frequency repetitive transcranial magnetic stimulation with transcranial direct current stimulation: evidence for homeostatic plasticity in the human motor cortex. J. Neurosci. 24, 3379–3385 10.1523/JNEUROSCI.5316-03.2004 15056717 PMC6730024

[B60] Sparing R. Dafotakis M. Meister I. G. Thirugnanasambandam N. Fink G. R. (2008). Enhancing language performance with non-invasive brain stimulation–a transcranial direct current stimulation study in healthy humans. Neuropsychologia 46, 261–268 10.1016/j.neuropsychologia.2007.07.009 17804023

[B61] Speisman R. B. Kumar A. Rani A. Pastoriza J. M. Severance J. E. Foster T. C. (2013). Environmental enrichment restores neurogenesis and rapid acquisition in aged rats. Neurobiol. Aging 34, 263–274 10.1016/j.neurobiolaging.2012.05.023 22795793 PMC3480541

[B62] Stagg C. J. Jayaram G. Pastor D. Kincses Z. T. Matthews P. M. Johansen-Berg H. (2011). Polarity and timing-dependent effects of transcranial direct current stimulation in explicit motor learning. Neuropsychologia 49, 800–804 10.1016/j.neuropsychologia.2011.02.009 21335013 PMC3083512

[B63] Stagg C. Nitsche M. (2011). Physiological basis of transcranial direct current stimulation. Neuroscience 17, 37–53 10.1177/1073858410386614 21343407

[B64] Thornton R. Light L. L. (2006). Language comprehension and production in normal aging, in Handbook of the Psychology of Aging, eds Birren J. E. Warner Schaie K. (Burlington, MA: Elsevier), 262–287

[B65] Vidal-Piñeiro D. Martin-Trias P. Arenaza-Urquijo E. M. Sala-Llonch R. Clemente I. C. Mena-Sánchez I. (2014). Task-dependent activity and connectivity predict episodic memory network-based responses to brain stimulation in healthy aging. Brain Stimul. 7, 287–296 10.1016/j.brs.2013.12.016 24485466 PMC4517193

[B66] Wirth M. Rahman R. A. Kuenecke J. Koenig T. Horn H. Sommer W. (2011). Effects of transcranial direct current stimulation (tDCS) on behaviour and electrophysiology of language production. Neuropsychologia 49, 3989–3998 10.1016/j.neuropsychologia.2011.10.015 22044650

[B67] Ziemann U. Siebner H. R. (2008). Modifying motor learning through gating and homeostatic metaplasticity. Brain Stimul. 1, 60–66 10.1016/j.brs.2007.08.003 20633369

